# Salvage Hepatectomy in Toxic Liver Syndrome as a Bridge to Orthotopic Liver Retransplantation—Case Series and Structured Narrative Review

**DOI:** 10.3390/jcm15103649

**Published:** 2026-05-09

**Authors:** Constantin Scholz, Evangelos Tagkalos, Jens Kamuf, Eva-Verena Griemert, Maria Hoppe-Lotichius, Beate K. Straub, Lukas Müller, Maximilian Moos, Tobias Huber, Janine Baumgart, Jens Mittler, Hauke Lang

**Affiliations:** 1 Department of General, Visceral and Transplant Surgery, University Medical Center of the Johannes Gutenberg University Mainz, 55131 Mainz, Germanymaria.hoppe-lotichius@unimedizin-mainz.de (M.H.-L.); tobias.huber@unimedizin-mainz.de (T.H.); janine.baumgart@unimedizin-mainz.de (J.B.); jens.mittler@unimedizin-mainz.de (J.M.); hauke.lang@unimedizin-mainz.de (H.L.); 2 Department of Anaesthesiology, University Medical Center of the Johannes Gutenberg University Mainz, 55131 Mainz, Germany; jens.kamuf@uni-mainz.de (J.K.); eva-verena.griemert@unimedizin-mainz.de (E.-V.G.); 3 Institute of Pathology, University Medical Center of the Johannes Gutenberg University Mainz, 55131 Mainz, Germany; beate.straub@unimedizin-mainz.de; 4 Department of Diagnostic and Interventional Radiology, University Medical Center Mainz, 55131 Mainz, Germany; lukas.mueller@unimedizin-mainz.de (L.M.); maximilian.moos@unimedizin-mainz.de (M.M.)

**Keywords:** orthotopic liver transplantation, anhepatic phase, salvage hepatectomy, toxic liver syndrome, primary non-functioning, Hyperlactataemia

## Abstract

**Background/Objectives:** Salvage hepatectomy with a prolonged anhepatic phase represents a potential life-saving strategy in patients with toxic liver syndrome (TLS) following orthotopic liver transplantation (OLT). Available evidence is limited to small case series. **Methods:** We retrospectively analyzed all patients undergoing OLT between 2014 and 2024 at a German transplant center. Among 497 patients, 33 required retransplantation, including four patients who underwent salvage hepatectomy due to refractory TLS. Clinical trajectories were analyzed descriptively. Additionally, a structured narrative review of the literature was performed. **Results:** Median age was 63 years (range 62–67), and median anhepatic time was 38.4 h (range 14.39–71.55). No patient died during the anhepatic phase. One patient died during retransplantation, and another during the postoperative course. Two patients survived long-term without neurological impairment. A literature review identified 30 relevant studies with reported mortality rates exceeding 50%. **Conclusions:** Salvage hepatectomy with subsequent retransplantation may serve as a life-saving bridge in selected patients with TLS. However, outcomes remain heterogeneous, and evidence is limited. This study provides detailed insights into perioperative physiological trajectories and clinical decision-making, which are insufficiently described in the current literature.

## 1. Introduction

Emergency salvage hepatectomy (SaH) is a rarely employed but feasible technique in patients experiencing hemodynamically relevant hepatic failure. While this approach is most commonly indicated in cases of primary non-function (PNF) with consecutive toxic liver syndrome (TLS) of the allograft following orthotopic liver transplantation (OLT), reports also exist of its application in severe liver trauma and eclampsia [1,2]. TLS is characterized by severe hepatic necrosis leading to cardiovascular shock and respiratory failure and hemodynamic deterioration, most likely caused by released toxic metabolites [2].

In such cases, removal of the toxic organ may be necessary to stabilize the patient.

An analysis of the United Network for Organ Sharing (UNOS) database, which examined 1364 patients who were listed for retransplantation (ReLT) between 2005 and 2020, demonstrated ~5.5% (*n* = 75) of these patients were already anhepatic prior to ReLT. Moreover, only approximately 50% of this subgroup survived beyond six months [3].

Given the rarity of this clinical scenario, larger studies are unlikely to be feasible. Furthermore, the maximum tolerable duration of the anhepatic phase as well as a more detailed characterization of perioperative physiological parameters in this extreme situation require further investigation.

In this case series, we present four patients who underwent emergency salvage total hepatectomy and investigated the clinical trajectory and outcome. Further, we performed a structured narrative literature review to identify similar studies in the context of salvage hepatectomy and compared outcomes.

## 2. Materials and Methods

All patients undergoing orthotopic liver transplantation (OLT) between January 2014 and 31 December 2024 were screened retrospectively from an institutional database. The decision to perform salvage hepatectomy was made in the event of rapid deterioration of hemodynamic stability combined with continuously rising lactate levels without adequate responsiveness to vasopressor therapy. A temporary portocaval shunt was created in all cases by performing an end-to-side anastomosis between the portal vein and the inferior vena cava. No specific caliber standardization was applied due to anatomical variability. During ReLT, the shunt was taken down and standard vascular reconstruction was performed. TLS was defined as progressive hepatic failure associated with refractory hemodynamic instability, rising lactate levels, and clinical evidence of graft non-function despite maximal supportive therapy, as determined in a multidisciplinary consensus.

Descriptive statistical analysis was conducted using the statistical program SPSS 29 (SPSS Inc., released 2014, IBM SPSS Statistics for Windows, Version 23.0, IBM Armonk, NY, USA). Figures illustrating clinical parameters were generated using GraphPAD Prism (Version 10.4.2).

**Figure 1:**
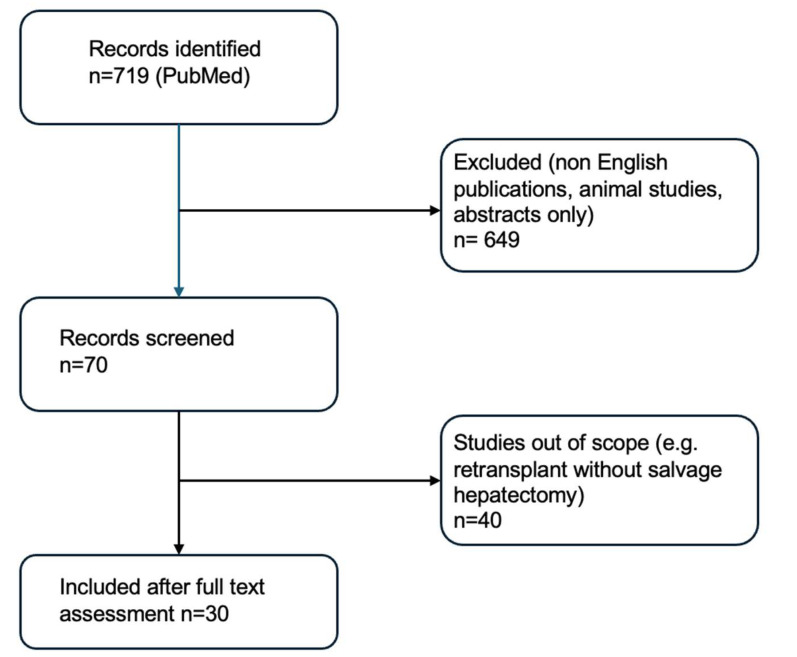
Flowchart demonstrating inclusion process of the literature for the structured narrative review.

Additionally, a structured narrative review was performed using MEDLINE (PubMed) to identify relevant studies. Given the rarity of the condition and the predominance of case reports, no formal systematic review methodology was applied (Appendix A). Only studies with confirmed prolonged anhepatic phase prior to ReLT were considered for inclusion. Studies analyzing outcomes after ReLT without previous SaH and prolonged anhepatic phase were excluded to avoid bias. The initial search yielded 719 articles. The following study types were considered for inclusion: case reports, case series, clinical studies, controlled clinical trials, guidelines, meta-analyses, observational studies, narrative reviews, randomized controlled trials, scoping reviews, and systematic reviews. Non-English publications and animal studies were excluded. After applying these criteria, 70 articles were screened for eligibility, of which 30 were ultimately included in the narrative literature review (retrospective studies: *n* = 4; case series: *n* = 10; case reports: *n* = 16) (Figure 1). Due to the predominance of case reports and small case series, no formal risk-of-bias or quality assessment tool was applied. The overall level of evidence was therefore considered low. Given the narrative design of this review, potential sources of bias—including selection bias and publication bias—cannot be excluded.

## 3. Results

Across all cases, the decision to proceed with SaH was based on a combination of clinical parameters. These included rapidly rising lactate levels (≥10 mmol/L in three patients), escalating vasopressor requirements indicating refractory shock, and clear evidence of graft non-function. In Case 4, despite lower peak lactate levels, the decision was driven by progressive graft necrosis and hemodynamic deterioration. All patients had failed prior surgical or interventional rescue attempts.

### 3.1. Cases

#### 3.1.1. Case 1

##### Indication—OLT

A 62-year-old male with hepatocellular carcinoma and underlying metabolic-associated steatotic liver disease (MASLD). MELD-Score: 8.

##### Operative Details

The OLT proceeded without complications using a classic approach (Vena Cava end-to-end anastomosis). Following reperfusion, the patient became hemodynamically unstable due to a hepatic artery thrombosis (HAT). Despite revision of the arterial anastomosis, blood flow in the peripheral liver segments remained insufficient.

##### ICU Course—Postoperative

**Figure 2:**
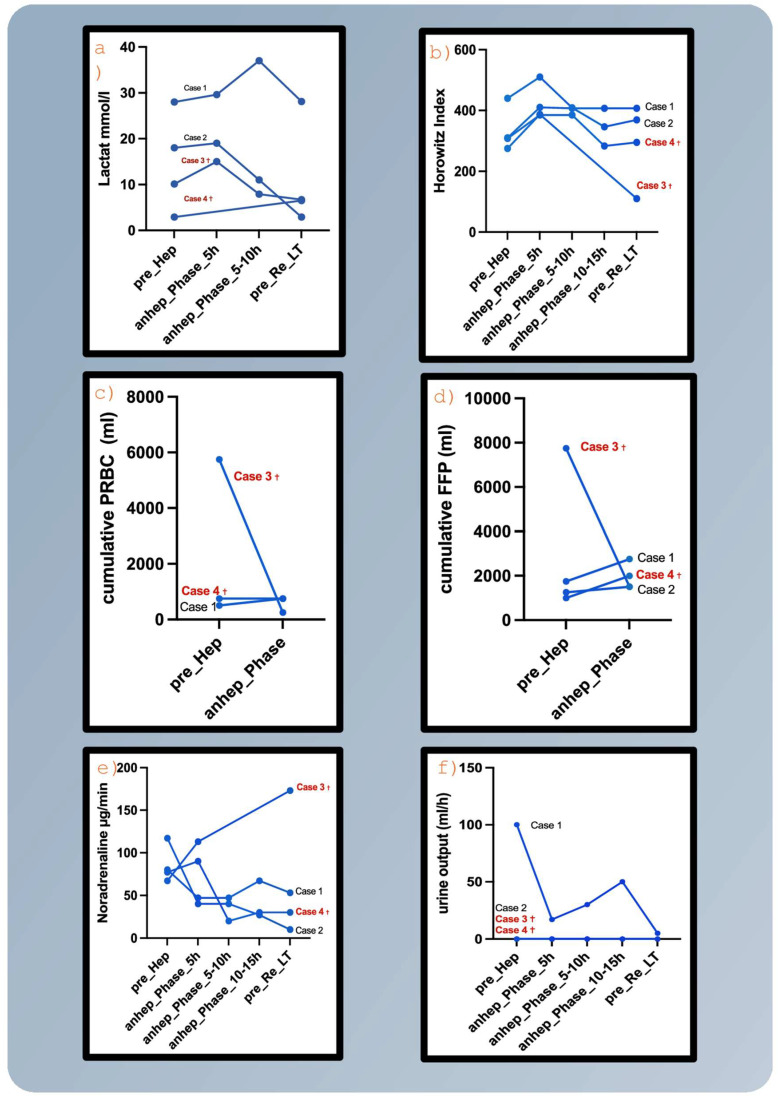
Clinical over time of (**a**) peak lactate levels, (**b**) Horowitz Index (Oxygenation Index), (**c**) cumulative amount of packed red blood cells (PRBCS), (**d**) fresh frozen plasma (FFP), (**e**) vasopressor (Noradrenaline) peak dosages and (**f**) urine output. pre_Hep, before salvage hepatectomy; anhep_Phase_5 h, first 5 h anhepatic phase; anhep_Phase_5–10 h, hour 5–10 h in anhepatic phase; anhep_Phase_10–15 h, hour 10–15 h in anhepatic phase; pre_Re_LT, before retransplantation. †, died.

In the ICU, the patient required further escalation of the vasopressor support, with a continuously rising lactate level and acid-base disturbances. Following progressive hemodynamic deterioration (Table 1, Figure 2), TLS was suspected. The patient was subsequently referred to the operating room for SaH.

##### Indication—Salvage Hepatectomy

TLS following PNF due to hepatic artery thrombosis.

##### ICU Course—Anhepatic Phase

During the anhepatic phase, which lasted 36 h and 41 min, the patient was stabilized hemodynamically with vasopressor support, fresh frozen plasma substitution and albumin (Figure 2). The patient remained intubated throughout the anhepatic phase. Subsequently, lactate levels declined (Figure 2).

##### Outcome

ReLT in standard technique was successfully performed. The patient was successfully weaned and extubated on POD 1. Five days after ReLT, the patient’s health status improved significantly, allowing for transfer to the surgical ward. Apart from a urinary retention (bladder voiding dysfunction) due to prostatic hyperplasia, no complications occurred. The patient was discharged after 23 days, is alive for 20 months and four weeks since liver transplantation.

#### 3.1.2. Case 2

##### Indication—OLT

A 67-year-old male with advanced alcohol-associated liver disease (ALD). MELD-Score: 25.

##### Operative Details

The liver was transplanted using the piggyback technique. After the release of vascular clamps for reperfusion, the liver remained unperfused. Despite the absence of portal vein and hepatic artery thrombosis, primary non-function (PNF) became evident (Table 1).

##### ICU Course—Postoperatively

TLS became evident by escalating vasopressor requirements and increasing lactate levels (Figure 2).

##### Indication—Salvage Hepatectomy

TLS following PNF.

##### ICU Course—Anhepatic Phase

In the ICU, CRRT was initiated, and coagulation management was optimized. The patient required vasopressor support (Figure 2). Transfusion products are shown in Table 1. The anhepatic phase lasted 38 h and 43 min.

##### Outcome

ReLT was performed without complications in standard technique. Extubation was feasible on POD 2. Following retransplantation, the patient experienced a complex postoperative course. Coagulation was severely impaired, requiring the administration of clotting factors and blood transfusions frequently. Due to multiple episodes of paroxysmal atrial fibrillation, amiodarone therapy was indicated. Additionally, the patient developed a pneumothorax and a large pleural effusion, both of which required drainage. Hemodialysis was continued due to persistently impaired renal function. On POD 23, the patient was transferred to the surgical ward. He was ultimately discharged on POD 82, fully recovered with a good graft function. The patient is alive 31 months and two weeks after OLT.

#### 3.1.3. Case 3

##### Indication—OLT

A 64-year-old male with recurrent hepatocellular carcinoma (HCC) in the setting of metabolic-associated steatotic liver disease (MASLD). The patient underwent repeated transarterial chemoembolization (TACE). MELD-Score: 12.

##### Operative Details

Following reperfusion, the liver became rigid and developed extensive reperfusion edema, accompanied by increased vasopressor requirements and elevated lactate levels up to 28 mmol/L. Right ventricular failure was suspected but not confirmed on transesophageal echocardiography (TEE). Subsequently, diffuse bleeding occurred, ultimately leading to a total graft failure and diagnosis of PNF.

##### ICU Course

The patient was transferred to the ICU with abdominal packing. Despite coagulation factor substitution, massive hemorrhage persisted, necessitating emergency relaparotomy and repeated abdominal packing.

##### Indication—Salvage Hepatectomy

TLS with massive hemorrhagic shock and hemodynamic instability.

##### ICU Course—Anhepatic Phase

In the ICU, pathological ST elevation on ECG with markedly elevated cardiac enzymes was observed. Echocardiography showed no abnormal myocardial movement, and thus, percutaneous coronary intervention or cardiac CT was not indicated. Despite the patient’s critical instability, a shared decision with the family was made to proceed with retransplantation.

##### Outcome

Intraoperatively, the patient’s hemodynamic status further deteriorated. Echocardiography revealed right ventricular failure. Given the severity of the condition, the surgery was terminated, and the patient died in the OR. The anhepatic phase lasted 14.39 h. The liver was re-allocated and successfully implanted.

#### 3.1.4. Case 4

##### Indication—OLT

A 62-year-old male with acute on chronic liver failure following ALD Child-Pugh C, complicated by portal vein thrombosis. MELD—Score: 37.

##### Operative Details

The liver was transplanted in piggyback technique. The reperfusion of the liver graft was prolonged but became sufficient after 20 min. The patient remained hemodynamically stable and was transferred to the ICU.

##### ICU Course—Postoperative

On POD 1, lactate levels and liver enzymes increased rapidly. Radiologic imaging confirmed portal vein thrombosis. The patient was taken to the OR for surgical thrombectomy; however, the liver was already partially necrotic, and thrombectomy using a Fogarty catheter was not successful. The procedure was terminated. A few hours later, the patient developed TLS.

##### Indication—Salvage Hepatectomy

TLS following PNF due to portal vein thrombosis.

##### ICU Course—Anhepatic Phase

After SaH, the patient was hemodynamically stable throughout the entire anhepatic phase, which lasted 71 h and 55 min.

##### Outcome

Eventually, ReLT was performed. Reperfusion occurred without incidents, and echocardiographic flow measurements showed a sufficient portal venous flow of 1000 mL/min (Table 1).

Despite the technically successful ReLT and good liver function, the patient required re-exploration due to active bleeding from Segment I and the gallbladder bed. The bleeding at the gallbladder bed was controlled with extensive electrocoagulation and packing. Ultimately, he demised due to extensive pulmonary embolism in the context of septic multi-organ failure. The patient survived for 1.9 months (58 days) following the anhepatic phase.

### 3.2. Recipient Characteristics

Between 1st January 2014 and 31st December 2024, 530 orthotopic liver transplantations were performed in 497 patients. Of the 33 patients requiring retransplantation, four developed toxic liver syndrome (TLS) necessitating SaH. All patients were male. Median age was 63 years (range 62–67).

The time interval between OLT and SaH ranged from 5.2 to 41 h (Table 1).

Median operation time was 94 min (range 80–159). Median anhepatic time was 38.4 h (range 14.39–71.55). Median peak ammonia was 152 µg/dL (range 73–163), and median peak lactate was 16.3 mmol/L (range 6.8–29.6). Despite elevated ammonia levels, no signs of permanent neurological sequelae or intracranial hypertension were observed in the surviving patients. Postoperative trajectories of catecholamine rates, oxygenation index (Horowitz Index), lactate levels, cumulative fresh frozen plasma and packed red blood cells are displayed in Figure 2. Donor characteristics of the OLT index are described in detail in Table 2.

## 4. Discussion

Salvage hepatectomy as a bridge to transplantation is a life-saving procedure in critical situations of several etiologies, where liver failure or uncontrollable hepatic hemorrhage poses a life-threatening risk to the patient [2]. Although emerging data suggest that prolonged anhepatic phases can be survived in selected cases, objective criteria for clinical decision-making in those patients are poorly defined [4,5,6,7].

We present a series of four patients who required SaH as a bridge to retransplantation. No patient died during the anhepatic phase itself. The reported deaths occurred either intraoperatively during retransplantation (Case 3) or during the postoperative course (Case 4). Temporary portocaval shunting was performed in all cases to prevent bowel ischemia following mesenteric venous congestion after SaH.

In Case 3, the decision to proceed with ReLT despite severe cardiac impairment reflects the complexity of decision-making in critically ill patients. In retrospect, markedly elevated cardiac biomarkers and evolving right ventricular failure likely represented relative contraindications. This case underscores the need for clearer criteria to identify patients unlikely to benefit from ReLT.

Management during the anhepatic phase also required careful adjustment of drug dosing due to absent hepatic metabolism. The use of hepatotoxic drugs was avoided whenever possible. Immunosuppressive therapy was initiated after retransplantation according to institutional protocols (methylprednisolone and calcineurin inhibitors). Continuous renal replacement therapy (CRRT) was used in all cases, contributing to metabolic stabilization and ammonia clearance, although detailed kinetics were not systematically assessed in this small cohort.

Despite the limited sample size, our study adds several clinically relevant aspects to the existing literature. First, we provide a detailed characterization of peri-anhepatic physiological trajectories, including lactate kinetics, vasopressor requirements, and transfusion burden, which are rarely systematically reported. Second, we describe real-world clinical decision-making leading to SaH, including timing and triggering parameters in critically ill patients. Third, we highlight the heterogeneity of outcomes despite similar initial presentations, emphasizing the need for improved risk stratification. Finally, our structured review contextualizes these findings within the existing low-level evidence base.

All patients in our series had TLS secondary to primary non-function (PNF; *n* = 4), characterized by rising lactate levels and hemodynamic instability. In two of these cases, hepatic artery thrombosis was also diagnosed. While one non-survivor exhibited persistently elevated lactate levels, the second non-survivor (Case 4) showed comparatively low peak lactate values, highlighting the inconsistency of lactate as a prognostic marker. While elevated lactate levels are commonly interpreted as a marker of disease severity, our findings demonstrate that lactate alone is insufficient to predict outcomes. Notably, Case 4 exhibited comparatively low peak lactate levels yet died due to late postoperative complications, indicating that lactate reflects acute metabolic stress rather than long-term outcomes. Therefore, lactate kinetics should be interpreted in conjunction with overall organ dysfunction and hemodynamic status rather than as an isolated prognostic parameter. However, given the small cohort size, this finding is exploratory only, and further studies are needed to investigate this association.

TLS is not precisely defined in the literature but is believed to result from extensive hepatic parenchymal necrosis, leading to cardiovascular disturbances, renal and respiratory failure, and a high lethality rate of up to 100% without salvage hepatectomy [8]. The release of vasoactive metabolites into circulation following hepatocyte death contributes to multiorgan failure, with liver removal is often the only option to stabilize hemodynamics and prolong life [9]. Ringe et al. were the first to establish clinical criteria for TLS, commonly characterized by acid-base disturbances, elevated lactate levels, and hemodynamic instability [10]. As demonstrated in several case reports and in our own cohort, hemodynamic stability frequently improves after the removal of the necrotic liver [11,12,13].

Primary non-function of the liver allograft is the most common cause of TLS. While there is no universally accepted definition of PNF, it is typically characterized by hypoglycemia, lactic acidosis, metabolic derangement, and encephalopathy, most of which were present in our patients [9].

Given the ongoing organ shortage and the increasing use of elderly donor organs, criteria for extended donor livers (ECD) have been introduced [14]. These marginal organs are associated with an increased risk of PNF. In our cohort, two patients with PNF received organs that met one or more criteria for ECD brain death donor livers (ECD-DBD) (CASE 2: cardiac arrest > 15 min, ALT > 140 U/L, macrosteatosis > 30%; CASE 4: cold ischemia time (CIT) > 12 h, ALT > 140 U/L) [15]. Notably, the donor in CASE 2 underwent 120 min of cardiopulmonary resuscitation and was subsequently placed on veno-arterial extracorporeal membrane oxygenation (VA-ECMO).

In addition to established extended-criteria donor (ECD) parameters, elevated donor gamma-glutamyl transferase (GGT) has recently been identified by Lai et al. as a potential risk factor for primary non-function (PNF) [16]. In our cohort, donor CASE 3 exhibited markedly elevated GGT levels (233 U/L), suggesting an additional marginal donor characteristic.

Our literature review revealed 30 articles published between 1988 and 2025 (retrospective studies: *n* = 4; case series: *n* = 10; case reports: *n* = 16) (Table 3). The overall lethality rate reported in the literature ranged between 0 and 80% (Table 3).

Although case reports describe anhepatic phases lasting up to 99 h, most series report a mean anhepatic duration between 10.1 and 48.8 h. In our series, the median anhepatic time was 38.4 h (range 14.39–71.55 h). The prolonged anhepatic time in our cohort can be attributed to the ongoing organ shortage, which is particularly pronounced in Germany, with one of the lowest organ donor rates in Europe (11.5 per 1,000,000 inhabitants) [36].

The best outcomes following prolonged anhepatic duration were reported by Cimeno et al., where three out of four patients survived the procedure with a mean anhepatic time of 48.8 h [6]. Notably, all patients in this cohort underwent portocaval shunting and, interestingly, received liver dialysis via the molecular adsorbents recirculating system (MARS). Although the authors acknowledge multiple confounding factors contributing to the ameliorated outcome, they suggest that MARS might help to create a “less hostile environment for retransplantation”, as clearance of cytokines, ammonia and lactic acid was measurably improved [6]. However, the availability of such technologies remains limited and their impact on survival is not yet clearly established.

The longest anhepatic time was reported by Laine et al., achieving 99 h in a 7-year-old female with Adams–Oliver syndrome, who survived the procedure and is alive 7 years later [5]. Notably, almost all cases of extremely prolonged anhepatic phases have been documented in either children or young adults (<35 years) [5,23,24,29]. Young adults are less likely to have several comorbidities or suffer from consequences of complex end stage liver disease, which is associated with severe deconditioning of the patient, co-existing impairment of several organ systems (renal, cardiovascular, respiratory), frailty and generally little physiological reserve [9]. In otherwise healthy individuals, certain metabolic compensatory mechanisms, such as renal gluconeogenesis, may temporarily mitigate complications like hypoglycemia. Lauritsen et al. demonstrated this phenomenon in their study on glucose homeostasis following hepatectomy in both pigs and humans [37].

Although no definitive futility threshold can be established, extremely prolonged anhepatic phases (>72 h) may be associated with increased risk of irreversible organ damage, particularly neurological injury. Given the rarity of this condition, multicenter registry-based analyses may represent the most feasible approach to generate clinically meaningful evidence. Notably, in contrast to the series reported by Ringe and Oldhafer prior to 2000, the number of published cases has declined over recent decades. This trend may reflect substantial advancements in immunosuppressive therapy and improved preoperative risk stratification for primary non-function (PNF), thereby reducing the incidence of cases requiring salvage hepatectomy [1,2,7,10]. However, there may be a bias due to underreporting of cases in the literature. As noted in the study referenced in the introduction, approximately 75 patients in the United States underwent an anhepatic phase between 2005 and 2020. In contrast, our literature review identified only four published reports from the U.S. since 2004, encompassing ten patients. This discrepancy strongly suggests that the true frequency of SaH cases followed by an anhepatic phase is likely much higher than currently documented. Underreporting may limit our understanding of the clinical course and outcomes associated with this high-risk intervention.

There are several limitations to this case study. Firstly, of course, the small sample size does not allow for definite conclusions or clinical decision-making. Interpretation of the results is further hampered by the risk of potential survivorship bias. However, larger studies may not be feasible due to the rarity of the event. Secondly, due to electronic data processing issues, cross-clamp time was not documented in all cases. Consequently, an approximate estimation of the anhepatic phase duration was made using the time interval between the end of hepatectomy and the completion of ReLT, rather than the more precise measurement from cross-clamp to reperfusion. This limitation may have led to a minimal underestimation of the actual anhepatic phase duration. Another issue concerns the interpretability of the literature. As almost solely case reports were identified during the literature search, statistical measures and estimates improving comparability between articles have been naturally lacking.

## 5. Conclusions

Salvage hepatectomy with subsequent retransplantation may represent a potential life-saving bridge in selected patients with TLS. The current evidence base, largely consisting of case reports, remains insufficient to draw definitive conclusions. Therefore, a global registry could help to create larger data sets and to identify underlying factors associated with beneficial outcome in this rare event.

## Figures and Tables

**Table 1:** Baseline characteristics of patients during anhepatic stage; LOS, length of stay (days); MELD Score, Model of End-Stage Liver Disease Score; OLT, orthotopic liver transplantation; SaH, salvage hepatectomy; CRRT, continuous renal replacement therapy.

Variables	Case 1	Case 2	Case 3	Case 4
Sex	male	male	male	male
Age	62	67	64	62
Weight (kg)	95	80	98	80
Height (cm)	180	178	175	165
Body Mass Index (kg/m^2^)	29.3	25.2	32.0	29.4
ASA	IV	III	II	IV
MELD Score	8	25	12	37
Indication Hepatectomy	PNF/TLS	PNF, TLS	PNF, TLS	PNF, TLS
Time OLT to SaH (h)	10.5	10.4	5.2	41
Operation Time Hepatectomy (min)	104	84	80	159
Anhepatic Phase (h)	36.41	38.43	14.39	71.55
Portocaval Shunt (yes/no)	yes	yes	yes	yes
Peak Lactate (anhepatic phase) (mmol/L)	13.6	19	29.6	6.8
Peak Ammonia (µg/dL)	152	163	152	73
Minimum Albumin (g/L)	16	25	28	26
CRRT	yes	yes	yes	yes
Death	no	no	yes	yes
Survival (months)	20	31	0	1.9
LOS (days)	23	82	1	58

**Table 2:** Donor characteristics of the orthotopic liver transplantation index. CPR, cardiopulmonary resuscitation; Na^+^, Natrium; SGPT, Serum Glutamate Pyruvate Transaminase; gGT, gamma-Glutamyltransferase; CIT, cold ischemia time.

Cases	Age	Cause of Death	CPR	Peak Na^+^ mmol/L	Peak SGPT U/L	Peak gGT U/L	CIT (h)
CASE 1	50	Traumatic subdural hemorrhage	No	148	43	56	10.20
CASE 2	38	Hypoxic brain death secondary to pulmonary embolism	120 min VA-ECMO	151	802	142	8.29
CASE 3	26	Hypoxic brain damage after pulmonary embolism	No	140	486	233	12.10
CASE 4	47	Subarachnoid hemorrhage	No	147	96	242	14

**Table 3:** Literature review. Abbreviations: HAT, hepatic artery thrombosis; PVT, portal vein thrombosis; PNF, primary nonfunctioning; TLS, toxic liver syndrome; HepA, hepatitis A infection; 7d-syndrome = 7-day syndrome; n.r., not reported.

Authors	Year	Country	Study Design	Sex	Age (Median/ Mean)	Underlying Condition	Indication Salvage Hepatectomy	Anhepatic Time (h)	Lethality
Ringe et al. [2]	1988	USA	Case series	Male (*n* = 2) Female (*n* = 2)	Median 28 years (Range 22–57)	Acute hepatitis, Budd–Chiari syndrome, autoimmune hepatitis, Klatskin tumor	n.r.	Mean 10.1 h	3/4
Bertholf et al. [17]	1992	USA	Case report	Female	11 years	HCC	HAT, PVT	34	0/1
Rozga et al. [18]	1993	USA	Case report	Female	18 years	-	ALF	14	0/1
So et al. [19]	1993	USA	Case series	Male (*n* = 1) Female (*n* = 1)	15 and 2 ½ years	Infantile oxalosis, hepatoblastoma	n.r.	26 h 48 h	0/2
Erhard et al. [20]	1993	Germany	Case report	Female	30 years	HELLP syndrome	TLS	20	0/1
Ringe et al. [10]	1993	Germany	Retrospective cohort study	Male (*n* = 17) Female (*n* = 15)	18 months–62 years	n.r.	TLS (27×) uncontrollable hemorrhage (6×)	Mean 16.4 h (0–41.29 h)	25/32
Henderson et al. [21]	1994	Australia	Case report	Female	23 years	Acute hepatitis B	TLS	12	0/1
Ringe et al. [7]	1995	USA	Case series	Male (*n* = 5) Female (*n* = 3)	Mean 22 years (Range n.r.)	-	Liver trauma (n = 8)	10.5 (Range n.r.)	6/8
Hunter et al. [22]	1995	USA	Case report	Female	28 years	-	Pre-eclampsia with hepatic rupture	13 h	0/1
Kim et al. [23]	1996	USA	Case report	Female	5 years	Myofibroblastic liver tumor	PNF	72 h	0/1
Hammer et al. [24]	1996	USA	Case report	Male	3 ½ years	Giant cell hepatitis	PNF	66 h	0/1
Oldhafer et al. [1]	1999	Germany	Case series	Sex n.r. (*n* = 20)	Mean 41 years (Range 13–66)	Budd–Chiari syndrome (2×), viral cirrhosis (6×), HCC (3×), hemangiosarcoma, CCC, trauma, graft failure, acute HepA, angiomatosis, alcohol-associated liver disease, cryptogenic cirrhosis, PSC	n.r. PNF	19.8 (6.58–72.50)	10/20
Bustamante et al. [25]	2000	Spain	Case series	Male (*n* = 4) Female (*n* = 1)	Median 55 years (Range 15–69)	ALF, viral cirrhosis, autoimmune cirrhosis, alcohol-associated liver disease, HCC	Uncontrollable hemorrhage (4×), liver trauma	Mean 19.4 (Range 16–24 h)	2/5
Dominguez-Fernandez et al. [26]	2001	Germany	Retrospective cohort study	Males (*n* = 3) Female (*n* = 3)	Mean 33 years	-	Liver trauma, intoxication, HELLP syndrome, TLS, after surgery	Mean 17.6 (Range n.r.)	5/6
Chiumello et al. [27]	2002	Italy	Case report	Male	29 years	-	Liver trauma	36 h	0/1
Guirl et al. [11]	2004	USA	Case series	Male (*n* = 4)	Range 29–31 years	Budd–Chiari syndrome, hepatitis C, PSC, cryptogenic liver cirrhosis	TLS (4×)	Mean 20 (Range 8–42)	0/4
Kodakat et al. [28]	2006	United Kingdom	Case report	Male	14 years	-	Liver trauma	35 h	0/1
Detry et al. [29]	2006	Belgium	Case report	Female	34 years	Cryptogenic cirrhosis	PNF	60 h	1/1
Detry et al. [30]	2007	Belgium	Case series	Females (*n* = 3)	Range 33–39 years	Viral cirrhosis PSC	HAT PVT (1×), uncontrollable bleeding, ALF	Mean 20 h (Range 17–24)	1/3
Ferraz-Neto et al. [8]	2008	Brazil	Case series	Male (*n* = 2) Female (*n* = 1)	Range 27–59 years	Alcohol-associated liver disease, Wilson’s disease, viral hepatitis	PNF, ALF, 7d-Syndrome	Mean 8.6 (4–14)	2/3
Lee et al. [31]	2010	Korea	Case report	Female	42 years	Klatskin tumor	ALF after resection	15 h	1/1
Arora et al. [4]	2010	USA	Case report	Male	46 years	Cryptogenic cirrhosis	PNF	67 h	0/1
Montalti et al. [13]	2010	Italy	Case series	Male (*n* = 2) Female (*n* = 2)	Mean 44 years (Range 30–56)	Viral hepatitis (2×), alcohol-associated liver disease, autoimmune hepatitis and HCC	PNF (2×), TLS, Tumor in allograft	Mean 19 h (Range 14–31.21 h)	1/4
Heneghan et al. [32]	2014	Ireland	Case report	Male	40 years	none	Heatstroke with rhabdomyolysis and hypoxic hepatitis	12	0/1
Mateos et al. [33]	2016	Ireland	Retrospective cohort study	Male (*n* = 4) Female (*n* = 2)	Mean 30.8 years (Range 20–47)	Viral hepatitis	Intoxication (3×) viral hepatitis, liver trauma, liver failure	15.5 h (Range 7–44)	0/6
Cimeno et al. [6]	2021	USA	Case series	Male (*n* = 2) Female (*n* = 2)	Mean 44.5 years (Range 32–71)	Viral hepatitis, PSC, cryptogenic cirrhosis, Wilson’s disease	HAT, PVT, PNF/TLS (2×)	48.8 (Range 33.6–55.8)	1/4
Singh et al. [34]	2022	USA	Case report	Male	67 years	Viral cirrhosis	PNF	27 h	0/1
Lurz et al. [12]	2022	Germany	Case report	Male	2 years	Biliary atresia	TLS	10.30 h	0/1
Laine et al. [5]	2023	Sweden	Retrospective cohort study	Male (*n* = 4) Female (*n* = 4)	Mean 31.6 years (Range 7 months-67)	Alcohol-associated liver disease (2×), biliary atresia (3x), PBC, viral cirrhosis, Adams–Oliver syndrome	PVT + HAT (4×) uncontrollable bleeding (3×) PNF	48.5 h (Range 14–99)	4/8
Coelho et al. [35]	2023	Brazil	Case report	Male	35 years	Autoimmune hepatitis	ALF	57 h	0/1

## Data Availability

The data generated and/or analyzed during the current study are available from the corresponding author on reasonable request.

## Abbreviations

The following abbreviations are used in this manuscript:

ALD	Alcohol-associated liver disease
CRRT	Continuous renal replacement therapy
FFP	Fresh frozen plasma
HAT	Hepatic artery thrombosis
MASLD	Metabolic dysfunction-associated steatotic liver disease
MELD	Model for end-stage liver disease
OLT	Orthotopic liver transplantation
PNF	Primary non-functioning
PRBCs	Packed red blood cells
ReLT	Retransplantation
SaH	Salvage hepatectomy

## Appendix A. Search Strategy

(“salvage hepatectomy” OR “total hepatectomy” OR “liver hepatectomy” OR “partial hepatectomy” OR “hepatectomy”)

AND

(“two-stage liver transplantation” OR “liver retransplantation” OR “liver transplantation” OR “liver graft failure” OR “primary liver nonfunction” OR “graft nonfunction”)

AND

(“anhepatic state” OR “anhepatic phase” OR “prolonged anhepatic state” OR “anhepatic management” OR “liver transplantation complications” OR “liver failure” OR “acute liver failure”)
